# Targeted massively parallel sequencing of autism spectrum disorder-associated genes in a case control cohort reveals rare loss-of-function risk variants

**DOI:** 10.1186/s13229-015-0034-z

**Published:** 2015-07-07

**Authors:** Anthony J. Griswold, Nicole D. Dueker, Derek Van Booven, Joseph A. Rantus, James M. Jaworski, Susan H. Slifer, Michael A. Schmidt, William Hulme, Ioanna Konidari, Patrice L. Whitehead, Michael L. Cuccaro, Eden R. Martin, Jonathan L. Haines, John R. Gilbert, John P. Hussman, Margaret A. Pericak-Vance

**Affiliations:** John P. Hussman Institute for Human Genomics, Miller School of Medicine, University of Miami, Miami, FL 33136 USA; Dr. John T. Macdonald Foundation Department of Human Genetics, Miller School of Medicine, University of Miami, Miami, FL 33136 USA; Department of Epidemiology and Biostatistics, Case Western Reserve University, Cleveland, OH 44106 USA; Hussman Institute for Autism, Baltimore, MD 21201 USA

## Abstract

**Background:**

Autism spectrum disorder (ASD) is highly heritable, yet genome-wide association studies (GWAS), copy number variation screens, and candidate gene association studies have found no single factor accounting for a large percentage of genetic risk. ASD trio exome sequencing studies have revealed genes with recurrent de novo loss-of-function variants as strong risk factors, but there are relatively few recurrently affected genes while as many as 1000 genes are predicted to play a role. As such, it is critical to identify the remaining rare and low-frequency variants contributing to ASD.

**Methods:**

We have utilized an approach of prioritization of genes by GWAS and follow-up with massively parallel sequencing in a case-control cohort. Using a previously reported ASD noise reduction GWAS analyses, we prioritized 837 RefSeq genes for custom targeting and sequencing. We sequenced the coding regions of those genes in 2071 ASD cases and 904 controls of European white ancestry. We applied comprehensive annotation to identify single variants which could confer ASD risk and also gene-based association analysis to identify sets of rare variants associated with ASD.

**Results:**

We identified a significant over-representation of rare loss-of-function variants in genes previously associated with ASD, including a de novo premature stop variant in the well-established ASD candidate gene *RBFOX1*. Furthermore, ASD cases were more likely to have two damaging missense variants in candidate genes than controls. Finally, gene-based rare variant association implicates genes functioning in excitatory neurotransmission and neurite outgrowth and guidance pathways including *CACNAD2*, *KCNH7*, and *NRXN1*.

**Conclusions:**

We find suggestive evidence that rare variants in synaptic genes are associated with ASD and that loss-of-function mutations in ASD candidate genes are a major risk factor, and we implicate damaging mutations in glutamate signaling receptors and neuronal adhesion and guidance molecules. Furthermore, the role of de novo mutations in ASD remains to be fully investigated as we identified the first reported protein-truncating variant in *RBFOX1* in ASD. Overall, this work, combined with others in the field, suggests a convergence of genes and molecular pathways underlying ASD etiology.

**Electronic supplementary material:**

The online version of this article (doi:10.1186/s13229-015-0034-z) contains supplementary material, which is available to authorized users.

## Background

Autism spectrum disorder (ASD) is a set of neurodevelopmental conditions diagnosed on the basis of a triad of symptoms: marked qualitative differences in social interaction, delayed or absent communication, and restricted interests or repetitive behaviors [1]. ASD is highly prevalent, occurring with a prevalence of approximately 1 in 68 8-year-olds in the United States [2], and confers significant costs to individuals and families. While the underlying physiological causes are largely unknown, twin concordance and family-based studies have shown ASD to be highly heritable, suggesting a strong underlying genetic etiology [3–5]. Therefore, candidate gene and common risk allele discovery has been a topic of much research, and more than 100 genes and 50 genomic loci have been implicated in autism by linkage analyses, copy number variation screens, and genome-wide association studies [6]. These candidates include single genes with mutations resulting in syndromes with ASD phenotypes [7–9], large cytogenetic aberrations [10], microduplications and microdeletions [11–15], and associated common alleles [16–20].

Overall, however, there has been limited replication across studies, demonstrating lack of support for the hypothesis that ASD is explained by common variants with strong to moderate effects. Instead, the common disease-rare variant (CDRV) hypothesis might better describe the underlying genetic architecture of ASD [21–23]. Identification of such rare genetic variants responsible for ASD risk has only recently been made possible with the application of massively parallel sequencing (MPS) technologies such as whole-genome sequencing (WGS), whole-exome sequencing (WES), and targeted region re-sequencing [24]. These techniques have been successful in the identification of the genetic causes of dozens of Mendelian disorders [25]. In ASD, which is phenotypically and genetically heterogeneous, WES studies thus far have focused primarily on the identification of de novo variation in family-based cohorts [26–32]. While this method has identified several potential ASD risk genes and mutations, each variant occurs at a low frequency in the population and the studies do not replicate well.

In contrast to this WES in trios approach, our strategy to detect rare functional variants in ASD is to apply MPS to genes within regions that have been implicated by common variant GWAS analysis in a large case-control cohort. We prioritized regions of association with GWAS-noise reduction (GWAS-NR) analyses of ASD datasets [16, 17] for follow-up sequencing, since disease-causing variants are likely not the common SNPs genotyped in GWAS. Sequencing of all exons of 837 RefSeq genes, in 2071 cases and 904 controls, revealed a significantly increased rate of stop gain/loss and splice altering loss-of-function (LOF) mutations, in subsets of candidate genes. Among the individual, LOF variants is a novel de novo premature stop gain in *RBFOX1* in an ASD case. Our unique strategy supports the hypothesis that LOF variants in previously implicated ASD genes, including *RBFOX1*, and novel genes underlying the complex genetic architecture of ASD.

## Methods

### Ethics statement

Individuals participating in this study have been collected over the course of several years, and ethical protocols with appropriate amendments have been approved. Individuals have been ascertained at the John P. Hussman Institute for Human Genomics (HIHG) at the University of Miami, Miller School of Medicine (Miami, FL), the University of South Carolina (Columbia, SC), and the Center for Human Genetics Research at Vanderbilt University (Nashville, TN). All participants were ascertained using protocols approved by the appropriate Institutional Review Boards, and the entire study falls under the University of Miami (UM) Institutional Review Board (IRB). This study was approved by the UM Medical Sciences IRB Committee B members: Ofelia Alvarez, M.D., Abdul Mian, Ph.D., Jose Castro, M.D., Jean Raymond Dauphin, O.D., Jean Jose, D.O., Norman Klein, J.D., Howard Landy, M.D., Stephen Richman, M.D., Eric Zetka, Pharm.D., and Liza Gordillo, M.S. In addition, samples from the Autism Genetic Resource Exchange (AGRE) and Simons Simplex Collection (SSC) were utilized. The use of AGRE and SSC samples is covered under the UM-IRB approved research protocol.

### Sample collection

A total of 2399 unrelated individuals affected with ASD were used in this study. Nine hundred fifty-six individuals were recruited using Institutional Review Board approved protocols through the John P. Hussman Institute for Human Genomics (HIHG) (912 individuals) at the University of Miami, Miller School of Medicine (Miami, FL) and the Center for Human Genetics Research at Vanderbilt University (43 individuals) (Nashville, TN). Criteria for inclusion for ASD-affected individuals were as follows: 1—between 3 and 21 years of age, 2—an ASD diagnosis using DSM-III-R or DSM-IV criteria [33] supported by the Autism Diagnostic Interview (ADI-R) [34], and 3—an IQ equivalent >35 or developmental level >18 months as determined by the Vineland Adaptive Behavior Scale (VABS) [35]. DNA was isolated from these individuals from whole blood collected via venipuncture.

Other ASD case DNA samples were included from the Autism Genetic Resource Exchange (14 individuals) [36] and the Simons Simplex Collection (1429 individuals) [37].

In addition, 1325 non-autistic control samples were acquired across two sites for this study. First, 525 individuals aged 4 to 21 years old were sampled through the HIHG. Participants were screened for eligibility by a questionnaire to determine whether the individual had been diagnosed with or had a parent or sibling with a developmental, behavioral, neurological, or other disability or physical conditions. If none of those conditions were present, parents of young children or the participants were informed and signed the informed consent and completed the Social Communication Questionnaire [38, 39] to screen for potential ASD. Three hundred twenty-six controls were part of a preterm birth study from cord blood collected at the Centennial Medical Center (Nashville, TN) from women aged from 18 to 40 years old with term pregnancies (>37 weeks gestation) and live, singleton births. The remaining 474 controls were obtained through the BioVu DNA repository at Vanderbilt [40].

### Targeted sequencing probe-set design

Regions to be targeted for sequencing in this study were determined using criteria previously described [16]. Briefly, the GWAS-NR algorithm was applied to two autism datasets and the top 5000 markers assorted into 2680 haplotype blocks [41] and 141 markers which were not in any block. Following statistical analysis using the truncated product method (TPM) of *p* values and a Monte Carlo simulation, 1535 genetic loci met a threshold of *p* ≤ 0.05. These regions were selected for follow-up sequencing in this study.

For target enrichment, we designed an Agilent SureSelect probe library using eArray web-based software (https://earray.chem.agilent.com/earray/) by bait-tiling 120-bp probes overlapping 60 bp. The targeted regions consisted of all exons of 837 RefSeq genes overlapping with blocks with *p* ≤ 0.05 TPM or nearest to significant intergenic blocks (Additional file 1). Nearest genes were chosen since GWAS-NR, by design, captures association from regions that may not be in strict linkage disequilibrium with a given SNP [16].

### Massively parallel sequencing of targeted regions

Samples were prepared following Agilent and Illumina protocols for in-solution SureSelect enrichment and sequencing. Briefly, 3 μg of genomic DNA, quantified using the Broad Range dsDNA Assay (Life Technologies, Grand Island, NY), is fragmented in a 96-microTUBE plate on the Covaris E210 (Covaris, Woburn, MA), followed by the verification of the peak size distribution ranging from 150 to 200 nucleotides using the LabChip GX (PerkinElmer, Waltham, MA).

The sheared DNA is then prepared for Illumina sequencing in 96-well plates using the Sciclone G3 NGS Liquid Handling Workstation (PerkinElmer, Waltham, MA). Capture hybridization begins with 500 ng of prepped sampled library, mixed with blockers, buffer, and biotinylated RNA probes during a 24-h incubation at 65 °C. Streptavidin beads bind the biotinylated RNA probes to extract the desired captured library. The bead-bound hybridized product is amplified and purified in 96-well plates using a Zephyr Compact Liquid Handling Workstation (PerkinElmer, Waltham, MA). The final product is then verified for a single peak size approximately 300 to 325 bp using the LabChip GX prior to sequencing.

All samples were indexed and multiplexed to run 7–11 samples per lane on the Illumina HiSeq 2000 (Illumina, San Diego, CA) for paired-end 2 × 100 bp sequencing. Raw sequence data was processed using the Illumina Run Time Analysis base calling pipeline, initially with v1.7 and later with v1.8.

### Data analysis

Subsequent processing was accomplished with an in-house sequencing data pipeline. This process includes alignment to the hg19 reference genome with the Burrows-Wheeler Aligner (BWA) [42], quality control with PICARD, and genotype calling performed with the Genome Analysis Toolkit (GATK) [43].

Sequencing quality control consisted of base quality score recalibration across flow cells and instruments as well as removal of duplicate reads resulting from PCR library amplification bias. For high-confidence genotype calling, we selected variants with a read depth greater than 8X, a call rate of at least 90 % across all samples, a log odds ratio under the trained Gaussian mixture model (VQSLOD) ≥ −3 and normalized, Phred-scaled likelihood of a reference genotype (PL) ≥200. The resulting alterations were annotated with SeattleSeq (http://snp.gs.washington.edu/SeattleSeqAnnotation137/index.jsp) and Polymorphism Phenotyping v2 (PolyPhen-2) (http://genetics.bwh.harvard.edu/pph2) [44]. LOF variants were defined as stop gains, stop losses, or splice site alterations [45].

### Sample quality control

From this total group of 3724 individuals, we excluded 6 samples with close relatedness or Mendelian inconsistencies identified by previously existing GWAS family data [17], 15 with underperforming sequencing metrics (less than 65 % of targeted bases covered at least 8X or call rates less than 90 % for all bases), and 8 with low (≤90 %) concordance with GWAS.

Racial and ethnic differences and population substructure have been demonstrated to have great impact on rare variant association analyses [46]; therefore, we set out to homogenize our sample cohort of 3695 using available Illumina 1M and 1M-duo genotyping array data [17]. We performed principal component analysis (PCA) using the “smartpca” script in EIGENSTRAT [47]. When available, we ran PCA on parents’ whole-genome genotyping data or, when parents were not available, on the individuals own. For those without genotyping data, we ran PCA with the sequencing data generated in this study. All participants who had both parents, or themselves when parental data was unavailable, fall within 2 standard deviations (SD) of the mean PC1 and PC2 values among our largest group of samples, clustering near the HapMap CEU population, were selected for further analysis. Additionally, all individuals with sequencing PCs within 2 SD of the mean PC1 and PC2 values of this genetically defined group defined above were also included. This analysis left 2975 European white individuals (2071 cases and 904 controls) for further analysis.

### Variant burden and association analyses

Comparison of the mean number of total, missense, and nonsense single nucleotide variants (SNVs) between cases and controls was done by unpaired *t* tests. Differential burden analysis of the number cases versus controls harboring loss-of-function variants was calculated by the Fisher exact test. We performed differential burden testing of loss-of-function genes across all genes and then restricted this to a list of strong ASD candidate genes using the curated SFARI gene database [48]. There were 45 genes with a gene score from SFARI that were included as the ASD candidate list.

We performed gene-based analyses testing association between targeted genes and ASD using the Optimized Sequence Kernel Association Test (SKAT-O) [49, 50] with default weights, adjusting for the first three PCs as covariates and correcting for gene size and linkage disequilibrium of variants within a gene. SNVs with differential case-control missingness *p* < 1.0 × 10^−6^ were excluded from analyses. This test was chosen because it is more powerful than collapsing and pooling methods in the presence of both risk and protective variants [50–53]. Analyses were run on several subsets of the variants including all and rare (minor allele frequency (MAF) ≤0.01) exonic, nonsynonymous, missense, predicted damaging, and LOF SNVs.

### Capillary sequencing

A subset of variants was analyzed by traditional capillary sequencing. Variant specific oligos were designed using the Primer3 (v. 0.4.0) (http://fokker.wi.mit.edu/primer3/input.htm) [54] and the UCSC genome browser (GRCh37/hg19). Sequencing reactions were performed with the Big Dye Terminator v3.1, sequenced on the Applied Biosystems 3730xl DNA Analyzer (Life Technologies, Grand Island, NY) and evaluated in the Sequencher software v4.10.1 (Gene Codes Corporation, Ann Arbor, MI).

## Results

### Sequencing output and quality control

Targeted MPS was performed to identify potential ASD risk alleles in 2071 ASD cases by comparing to 904 non-autistic controls in GWAS-NR prioritized regions. We generated 30.3 ± 9.7 million passing filter Illumina HiSeq 2 × 100 bp reads per individual sample with only 8.6 ± 5.9 % duplicate reads removed to avoid amplification artifacts. Of the remaining reads, 96.8 ± 1.4 % aligned to the human genome and 81.2 ± 10.2 % aligned to the 29.1 Mb “near target” region, consisting of bases covered by targeting probes and their 200-bp flanks. This highly efficient targeting, sequencing, and alignment lead to average depth coverage of 78.1 ± 25.9 times of the near target bases and 87.6 ± 5.6 % of targeted bases covered at least 10X.

### Genotype calling and variant discovery

Overall, we identified 545,916 genomic positions at which at least one of the 2911 individuals analyzed had a non-reference genotype call, meeting our genotype calling quality criteria. Of those, 231,945 SNVs (56.2 %) were not previously annotated in dbSNP137. Genotype call quality and sample matching integrity was determined by comparison of SNP genotyping data available for most samples [17, 18]. The SNV calls between targeted sequencing and genotyping arrays at over 30,000 markers had an average concordance of 99.3 ± 0.01 %.

### Functional annotation of variants

We focused our analyses on SNVs affecting only coding exons of the targeted genes to best identify functional variation. We identified 25,966 such SNVs in our cohort including 16,330 (62.9 %) SNVs were not previously reported in dbSNP134. We employed publically available databases to annotate each variant, including PolyPhen2 damaging predictions. When multiple isoforms were identified, the most deleterious SNV was included. To allow for comparison between cases and controls, we normalized the number of variants correcting for differences in sequence coverage and missing data. This normalization procedure gave extremely consistent rates of variation in cases and controls. We identified no difference between cases and controls in the overall number of SNVs nor in subsets of missense, predicted damaging, or nonsense and splice site variants (Table 1).

**Table 1:** Categories of coding variation per individual

	Cases	Controls
Total variants	23,756 ± 836	23,749 ± 797
Coding	765 ± 62	760 ± 64
Missense	307 ± 30	305 ± 31
Damaging	85 ± 12	84 ± 13
Nonsense/splice (loss-of-function)	3 ± 1.4	2.5 ± 1.8

All coding variant passing quality control filters were annotated and categorized by ANNOVAR into these categories

### Gene-based rare variant association

Because we did not identify a global difference in the number of SNVs between cases and controls, we tested the hypothesis that sets of protein-coding SNVs in genes would be associated with ASD using gene-based rare variant set tests with SKAT-O. We restricted our analyses to rare SNVs (MAF ≤0.01) and identified nominal association (*p* ≤ 0.05) with 16, 19, and 25 genes when examining exonic, nonsynonymous, and damaging SNVs, respectively, (Table 2). However, no gene was significant in any SKAT-O analysis after correction for multiple gene testing (corrected *p* = 9.5 × 10^−5^).

**Table 2:** Gene-based SKAT-O results for genes in selected categories with *p* < 0.05

Exonic (524 genes)		Nonsynonymous (499 genes)		Damaging (504 genes)	
Gene	*p* values	Gene	*p* values	Gene	*p* values
*CACNA2D1*	0.000478745	*PLA2G6*	0.00959325	*ERMAP*	0.00955634
*GNPTAB*	0.004651201	*CDC42EP4*	0.010211946	*SLC17A9*	0.011358674
*BICD1*	0.008804534	*SLC17A9*	0.010656919	*KIAA1274*	0.01243212
*PPM1H*	0.011408084	*AMIGO2*	0.016812058	*ZNF519*	0.017563864
*DDO*	0.0124426	*GNPTAB*	0.018063006	*ANKRD22*	0.020850511
*DGKG*	0.015419571	*OSGIN2*	0.019535871	*PA238*	0.021527527
*PKP4*	0.0213883	*ANKRD22*	0.020850511	*CDC42EP4*	0.023737296
*PARVA*	0.022742454	*PAX8*	0.021527527	*PTPRK*	0.023809113
*BACH2*	0.026375169	*ERMAP*	0.021748476	*PDK4*	0.02412783
*LOC158381*	0.026815002	*PDK4*	0.024493537	*OSGIN2*	0.02476071
*SLC17A9*	0.032466663	*SPON1*	0.026942389	*FMN2*	0.026304563
*IL17RA*	0.035317441	*NTRK3* ^a^	0.027348276	*SPON1*	0.026942389
*QBRICK*	0.035922397	*DGKI*	0.030212727	*SLITRK5*	0.028283659
*NRXN1* ^a^	0.040563396	*FAM19A4*	0.030428212	*DGKI*	0.028626935
*KCNH7*	0.043770294	*THAP2*	0.042761309	*SLC16A12*	0.030211966
*TG*	0.047652525	*C18orf22*	0.044978318	*FAM19A4*	0.030428212
		*FGFR2*	0.046420494	*SLC24A2*	0.031130372
		*SPANXN3*	0.047977446	*DDO*	0.032382611
		*KCNH7*	0.048279279	*TG*	0.035323522
				*KIAA0914*	0.036561743
				*ZNF396*	0.038298063
				*THAP2*	0.046033231
				*C18orf22*	0.046801921
				*PDZD2*	0.046866496
				*LASS6*	0.047604531

Rare variants (MAF <0.01) within genes underwent gene-based association testing by SKAT-O. All genes with nominal SKAT-O *p* values <0.05 are represented

^a^Gene is a potential ASD candidate gene in the SFARI Gene Database (https://gene.sfari.org/autdb/)

### Individual variant prioritization

Since the autism phenotype is highly variable and the numbers of candidate genes are many, rare SNVs found in only one or a few individuals may be clinically relevant by leading to an increased risk or causation of the disorder. We employed a two-tier strategy to identify individual SNVs or genes contributing to autism etiology. First, we identified genes with homozygous or compound heterozygous rare (MAF ≤0.01 for each variant allele), damaging (as predicted by SIFT or PolyPhen2) SNVs. Among all individuals, a total of 47 genes were affected by at least two rare damaging variants in the same individual (Additional file 2). Among the genes with two hits uniquely in cases were three candidate genes linked to autism by a literature-curated database*, DMD*, *SYNE1*, and *TBL1X* and two genes implicated in schizophrenia (*GRIK4*) and intellectual disability (*PQBP1*). Overall, there are significantly more ASD cases (144/2071) than controls (26/904) carrying genes with two rare predicted damaging SNVs (Fisher’s exact *p* = 0.0001).

The second strategy identified rare SNVs (MAF ≤ 0.01) causing LOF. These alterations cause nonsense premature stops or stop losses and splice site changes. Among our 2975 individuals, we identified 464 rare LOF variants (Additional file 3). Across all these variants, there was no overall enrichment of LOF in ASD cases (Fisher’s exact *p* = 0.325). However, when restricting the gene list to only those that have been implicated previously in ASD, there is a significant enrichment of loss-of-function variants in cases with 27 cases carrying such variants compared to only four controls (Fisher’s exact *p* = 0.032).

### Inheritance of loss-of-function SNVs

**Fig. 1:**
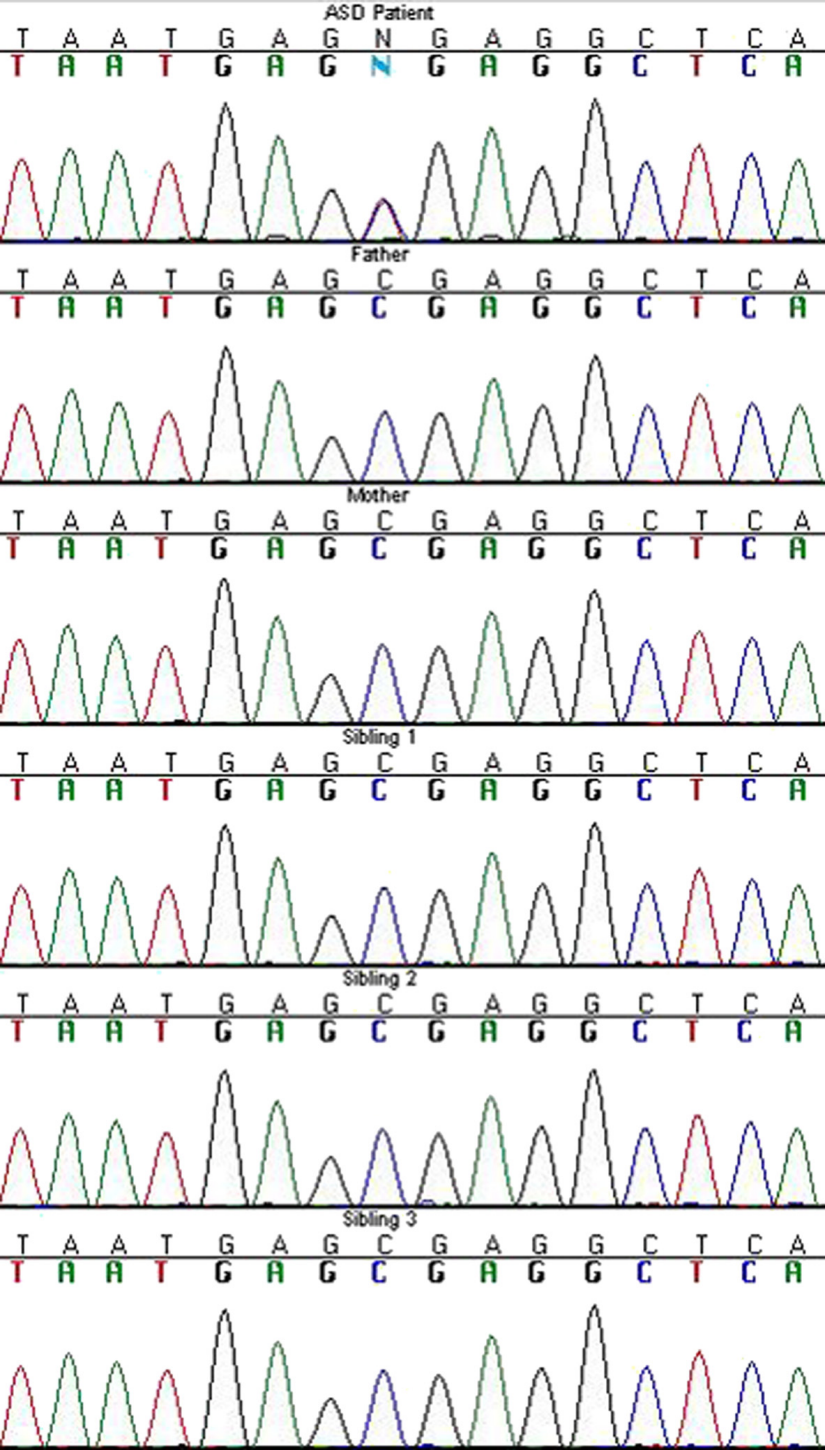
Sanger sequencing of the loss-of-function de novo variant in *RBFOX1*. The patient in whom the *RBFOX1* premature stop variant was identified along with parents, and three unaffected siblings were sequenced using standard Sanger capillary sequencing. The ASD patient has a *C*/*T* genotype (*N*) while all other family members are *C*/*C*

Since de novo variations in protein-coding genes have been recently shown to be a strong risk factor for autism, we determined the inheritance status of the LOF SNVs in seven ASD candidate genes and an additional ten LOF SNVs in genes with putative neuronal function (Table 3). We performed standard capillary sequencing of the individual in which the SNV was identified along with both parents and available siblings, if any. All 17 variants were validated. Of the 17 variants examined, one, a premature stop codon in *RBFOX1*, was de novo (Fig. 1), 13 were maternally inherited, and three were paternally inherited. Two of the variants, premature stop alterations *GPR110* and *DOCK1*, were identified in an individual from a family with multiple affected individuals. The variant in *GPR110* did not segregate with the ASD, but the LOF SNV in *DOCK1* is identified in the affected pro-band and ASD-affected mother and not an unaffected sibling.

**Table 3:** Case unique loss-of-function variants selected for Sanger validation

Position	Gene	Coding nucleotide	Amino acid
chr1:146737632	*CH1DL*	c.C169T	p.R57X
chr2:32434592	*SLC30A6*	c.C625T	p.R209X
chr3:154139052	*GPR149*	c.C1399T	p.Q467X
chr4:187517886	*FAT1* ^a^	c.C481T	p.R161X
chr5:75866423	*IQGAP2*	c.C322T	p.R108X
chr5:108294935	*FER* ^a^	c.C1018T	p.R340X
chr5:148407104	*SH3TC2*	c.G832T	p.E278X
chr6:46988468	*GPR110*	c.C610T	p.R204X
chr6:102483442	*GRIK2* ^a^	splice	splice
chr6:116288798	*FRK* ^a^	c.C715T	p.R239X
chr6:152690106	*SYNE1* ^a^	splice	splice
chr10:34400099	*PARD3*	splice	splice
chr10:129183056	*DOCK1*	c.G3747A	p.W1249X
chr13:109859019	*MYO16* ^a^	c.G5478A	p.W1826X
chr15:58004192	*GCOM1*	c.C1315T	p.R439X
chr16:7637291	*RBFOX1* ^a^	c.C517T	p.R173X
chr22:17588636	*IL17RA*	c.C1065G	p.Y355X

Rare loss-of-function variants in genes with presumed neuronal genes were subjected to Sanger validation and familial segregation testing. Coordinates are based on the hg19 human genome reference build

^a^Gene is a potential ASD candidate gene in the SFARI Gene Database (https://gene.sfari.org/autdb/)

## Discussion

ASD has few common variants known to impact genetic risk leading to the hypothesis that rare variants with larger effects in candidate genes are contributing to the phenotype. Since the objective of GWAS-NR is to identify regions of association for follow-up analysis [16], our study set out to identify such rare variants specifically in previously associated candidate genes. Similar approaches have been successful at identifying the contribution of rare variants in other complex diseases such as type II diabetes [55], hypertriglyceridemia [56], and inflammatory bowel disease [57, 58].

The majority of sequencing studies in ASD thus far have been whole-exome sequencing in large trio-based cohorts [26–32]. These efforts are designed specifically to identify de novo variants of high effect and have identified several recurrent de novo loss-of-function changes as strong risk factors for ASD. While the current study was not designed for identification of de novo variants, we undertook a two-pronged approach to identify individual loss-of-function variants that could have major effects. Firstly, we identified a statistically significant increase in the number of genes with at least two rare predicted damaging alterations in the same individual in ASD cases compared to controls (*p* = 0.0001). Secondly, we found an excess of LOF mutations in ASD candidate genes in our cases (*p* = 0.032). Overall, this suggests that carrying two damaging hits or LOF in previously identified candidate genes is a risk factor for ASD. This is similar to previous WES studies specifically identifying two-hit and LOF mutations as risk factors in ASD [30, 59].

Among these loss-of-functions, we identified a de novo premature stop variation in the splice regulation factor *RBFOX1*. This is an excellent candidate gene as several genomic lesions including microdeletions [11, 60, 61] and rare missense mutations [62] have been reported in ASD cases in *RBFOX1.* Additionally, its role as a splice regulatory factor [63] was highlighted in a transcriptome study where aberrant splicing of *RBFOX1*-dependent alternative exons in the brains of ASD patients was identified [64]. Targets of RBFOX1 splice regulation are enriched for genes involved in neuronal excitation [65] and cytoskeletal reorganization [64]. This study offers the first evidence of a de novo or LOF sequence variant in *RBOFX1* present in an ASD patient.

While the presence of a premature stop or splice altering variants does not necessarily indicate a molecular loss-of-function, the overall analysis points to genes involved in several underlying physiological mechanisms that support a role for excitatory neurotransmission and altered neurite outgrowth and guidance in ASD. In the analysis of genes with two damaging mutations unique to ASD cases, we identified two genes involved in the glutamatergic signaling pathway, the metabotropic glutamate receptor 3, *GRM3*, and glutamate receptor, ionotropic, kainate 4, *GRIK4.* Common variants in *GRM3* have been shown to have association with ASD [66] while rare CNVs in *GRIK4* have been implicated [11], and both genes have evidence for association in bipolar disorder [67] and schizophrenia [68, 69]. *IL1RAPL2* is a gene related to ASD candidate gene *IL1RAPL1*, which regulates the formation and stabilization of glutamatergic synapses [70], and rare mutations have been implicated in ASD and neurodevelopmental phenotypes [71, 72]. Neuronal adhesion and guidance molecules are also implicated. *SEMA6A* has not been directly implicated in ASD or other neuropsychiatric disorders, but mouse mutants demonstrate anatomical abnormalities in limbic and cortical cellular organization [73] and axon guidance [74]. Finally, polyglutamine-binding protein 1 (*PQBP1)* has been implicated as a splice factor necessary for proper neurite outgrowth [75], and rare mutations in *PQBP1* have also been implicated in X-linked mental retardation [76].

Likewise, a commonality of function can be identified among the loss-of-function SNVs. [45]. There are 199 genes with LOF variants uniquely in cases, including 17 in genes previously linked to ASD. Among the most intriguing candidates are two stop inducing SNVs in the cell polarity regulator *FAT1* and two stop and one splice SNV in the nuclear envelope protein *SYNE1* which have previously been implicated by de novo missense variants in ASD cases [26, 29], though the variants in this study were maternally inherited. The glutamate receptor *GRIK2* has been associated with ASD and neurodevelopment several studies [11, 66, 77, 78], and we identify a premature stop and two splice change SNVs. We detected two stop and one splice SNV in the well-recognized ASD candidate gene *NRXN1* [79–82].

Taking into account all variants in this study, no differences in global burden of variation were identified between cases and controls reflecting the findings of whole-exome studies in which the rate of mutations is similar in cases compared to unaffected controls or siblings [26, 28, 29, 83]. Additionally, no individual coding variants were significantly associated with ASD. As such, we applied rare variant association testing using SKAT-O but did not identify any gene with a statistically significant association of rare variants. Despite having one of the largest reported sample sizes for an ASD case-control sequencing study, it has been suggested that even larger sample sizes on the order of tens of thousands of cases will be required to identify associations of rare variants with complex disease [84]. However, investigation of the nominally significant genes can be informative for potential roles of several genes in ASD risk.

For example, when observing genes with nominal association between ASD and sets of rare exonic variants, the most significant gene is *CACNA2D1* (Calcium Channel, Voltage-Dependent, Alpha 2/Delta Subunit) (*p* = 0.00047). *CACNA2D1* regulates influx of calcium ions into the cell upon membrane polarization and Mutations in a related gene (*CACNA1C*) are known to cause Timothy syndrome which includes neurological and developmental deficits and often autism symptoms [85]. In addition, rare exonic and nonsynonymous variants in the potassium channel *KCNH7* showed nominal association (*p* = 0.043). Variants in this gene have been previously linked to bipolar disorder [86], schizophrenia [87], and developmental delay [88]. Rare exonic variants in the well-established ASD and schizophrenia candidate gene *NRXN1* [79–81] show nominal association (*p* = 0.041). Such pleiotropic relationships between variants conferring ASD risk and other neuropsychiatric disorders have been documented [89, 90]. Overall, the nominally significant gene sets point toward synaptic function as critical in risk to ASD, and an increase in sample size and collaboration across multiple datasets may increase our power to detect significant associations with these or other potential candidate genes.

In conclusion, previous studies of the genetic causes of ASD have implicated hundreds of potential loci with none fully explaining the extent of genetic risk. Therefore, it is a necessity to identify the many rare variants in numerous genes contributing to the disorder using existing GWAS data to prioritize regions of ASD-specific susceptibility variants and then find underlying rare risk variants using recently developed massively parallel sequencing technologies. This has the distinct advantage of reduced cost, ease of multiplexing, and specific information content as compared to whole-exome sequencing studies.

## Conclusions

We have used this approach to arrive at three observations. First, our evidence is suggestive that accumulation of rare variants in synaptic genes, including *CACNAD2*, *KCNH7*, and *NRXN1*, is associated with ASD, but independent support from future sequencing studies will be necessary to statistically support this idea. Second, we identified an over-representation of two damaging hit across all genes and LOF mutations in ASD candidate genes as a risk factor for ASD and implicate damaging mutations in glutamate signaling receptors and neuronal adhesion and guidance molecules. Finally, we provide supporting evidence of de novo coding mutations and the role of *RBFOX1* dysfunction as a potential risk factor for ASD. These observations highlight the heterogeneity of the genetic etiology of ASD but point toward a convergence of pathways and mechanisms which underlie the complex phenotype.

## Additional files

Additional file 1:
**Genes overlapping or nearest GWAS-NR association peaks.** File includes gene name and hg19 genomic coordinates of the canonical transcript of each targeted gene. Additional file 2:
**Genes with at least two rare (MAF < 0.01), damaging SNVs.** File includes the gene name, total count of rare damaging variants in any individual, and the count of cases and controls with at least two. Additional file 3:
**Rare loss-of-function variants.** Table includes position, base change, and frequency of each of the 464 loss-of-function variants identified in our cohort.

## Abbreviations

ASD: autism spectrum disorder
BWA: Burrows-Wheeler Aligner
GATK: Genome Analysis Toolkit
GWAS-NR: genome-wide association study-noise reduction
LOF: loss-of-function
SKAT-O: optimized sequence kernel association test
SNV: single nucleotide variant
